# Enhanced High-Temperature Cycling Stability of Garnet-Based All Solid-State Lithium Battery Using a Multi-Functional Catholyte Buffer Layer

**DOI:** 10.1007/s40820-024-01358-9

**Published:** 2024-02-19

**Authors:** Leqi Zhao, Yijun Zhong, Chencheng Cao, Tony Tang, Zongping Shao

**Affiliations:** WA School of Mines: Minerals, Energy and Chemical Engineering, Curtin University, Perth, WA 6102 Australia

**Keywords:** Solid-state battery, Cathode electrolyte interlayer, Flame-retardant additive, Cycling stability, Interfacial stability

## Abstract

The pursuit of safer and high-performance lithium-ion batteries (LIBs) has triggered extensive research activities on solid-state batteries, while challenges related to the unstable electrode–electrolyte interface hinder their practical implementation. Polymer has been used extensively to improve the cathode-electrolyte interface in garnet-based all-solid-state LIBs (ASSLBs), while it introduces new concerns about thermal stability. In this study, we propose the incorporation of a multi-functional flame-retardant triphenyl phosphate additive into poly(ethylene oxide), acting as a thin buffer layer between LiNi_0.8_Co_0.1_Mn_0.1_O_2_ (NCM811) cathode and garnet electrolyte. Through electrochemical stability tests, cycling performance evaluations, interfacial thermal stability analysis and flammability tests, improved thermal stability (capacity retention of 98.5% after 100 cycles at 60 °C, and 89.6% after 50 cycles at 80 °C) and safety characteristics (safe and stable cycling up to 100 °C) are demonstrated. Based on various materials characterizations, the mechanism for the improved thermal stability of the interface is proposed. The results highlight the potential of multi-functional flame-retardant additives to address the challenges associated with the electrode–electrolyte interface in ASSLBs at high temperature. Efficient thermal modification in ASSLBs operating at elevated temperatures is also essential for enabling large-scale energy storage with safety being the primary concern.

**Supplementary Information:**

The online version contains supplementary material available at 10.1007/s40820-024-01358-9.

## Highlights

- Thermally stable catholyte buffer layer was fabricated via incorporating a multi-functional flame-retardant triphenyl phosphate additive into poly(ethylene oxide).
- The optimized catholyte buffer layer enabled thermal and electrochemical stability at interface level, delivering comparable cycling stability of garnet-based all solid-state lithium battery, i.e., capacity retention of 98.5% after 100 cycles at 60 °C, and 89.6% after 50 cycles at 80 °C.
- Exceptional safety performances were demonstrated, i.e., safely cycling behavior at temperature up to 100 °C and spontaneous fire-extinguishing ability.

**Supplementary Information:**

The online version contains supplementary material available at 10.1007/s40820-024-01358-9.

## Graphical abstract

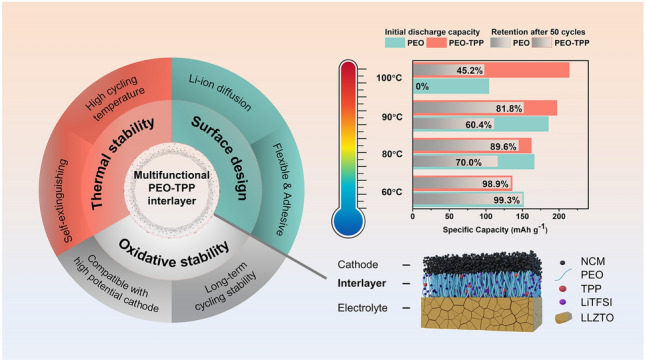

## Introduction

Conventional lithium-ion batteries (LIBs) have transformed the energy storage industry significantly. However, their use, in particular at large scale, is associated with safety concerns due to the presence of flammable liquid electrolytes. All-solid-state LIBs (ASSLBs) have emerged as a promising alternative, offering enhanced safety and compatibility with high-capacity cathodes [1]. Nonetheless, ASSLBs face several new challenges, including lower ionic conductivity and poor cycling performance compared to their liquid counterparts. Such challenges are not only limited to the electrolyte but also encompass the interfaces between the electrodes and electrolyte [2, 3].

Garnet electrolytes are a class of solid-state electrolytes based on lithium-stuffed garnet crystal structures, typically represented as Li_7_La_3_Zr_2_O_12_ (LLZO) [4, 5], which have received considerable attention because of their modest conductivity at room temperature (RT) and favorable chemical stability. The main concerns in the practical use of LLZO-based ASSLBs include insufficient conductivity at low temperature and unstable anode-electrolyte interface and cathode-electrolyte interface. Insufficient conductivity will cause poor rate performance and capacity, while electrodes-garnet electrolyte interfaces instability causes poor interfacial contact and limited ionic conductivity, thereby impeding efficient charge transfer. On the other hand, dendrite formation, particularly in lithium metal anodes, is another critical issue for the anode, as dendrites can breach the electrolyte, leading to safety hazards and short circuits [6]. Additionally, thermodynamically unstable interfaces can lead to undesirable side reactions, depleting active materials and decreasing overall device performance [7].

To address above-mentioned issues, various strategies were employed, including surface modification, electrolyte optimization, interlayer integration, creation of composite Li anode and structural design within electrolyte [8]. Cathode-garnet electrolyte interfaces require particular attention. Volume expansion and contraction-induced stress occurs during charge and discharge cycles, inducing strain at the cathode-electrolyte interface which can potentially cause interface delamination, compromising battery stability. In some studies, lithium-conducting interlayers were incorporated to enhance interface contact and improve conductivity by facilitating smooth lithium-ion transfer and reducing interfacial resistance, in particular the use of Li_3_PO_4_ [9] can help weaken the space charge layer and provide binding effect for cathode and electrolyte. In some other studies, composite cathodes were used to reinforce the interface contact, which comprises of cathode active material and small amount of garnet, e.g., LiNi_0.8_Mn_0.1_Co_0.1_O_2_ (NCM811) + Li_6.4_La_3_Zr_1.4_Ta_0.6_O_12_ (LLZTO) [10] composite, to facilitate compact contact without incurring side reactions from new substance. Fabrication of garnet 3-D structure was also reported to be effective as Li^+^ conductive network, by integrating conductive polymer such as poly(ethylene oxide) (PEO) [11]. Furthermore, electrolyte additives were introduced to stabilize the interface and suppress undesired reactions, forming protective layers or passivating films that reduce active material consumption and enhance device performance [12–14].

Recently, solid-state electrolytes (SSEs) incorporating both polymers and inorganic materials have emerged, involving the combination of a polymer-based electrolyte with an active inorganic filler [15]. Despite improvements in the mechanical strength of the composite electrolyte compared to the original solid polymer electrolyte (SPE), challenges such as dendrite formation and side reactions on the Li/Polymer interface remain [16]. Alternatively, polymers have been extensively utilized as wetting agents between cathode and electrolyte to improve physical contact while preserving the mechanical property of garnet electrolyte, i.e., incorporation of polymer as cathode electrolyte (catholyte) interlayer [17, 18]. PEO has long been considered a promising choice for this purpose, owing to PEO's capability for high Li^+^ solvation, facile fabrication, and cost-effectiveness [19]. This approach is expected to play a crucial role in enhancing ionic conductivity and addressing interfacial compatibility issues within and between SSEs and electrodes. However, PEO is susceptible to decomposition at high voltages and high temperatures. Polymer matrix consisting of poly(vinylidene fluoride-hexafluoropropylene) (PVdF-HFP) [20] and PEO blends have been tried as catholyte interlayer with favorable cycling stability. The combination of PVDF-HFP and PEO can offer enhanced anti-oxidation capability with a wider electrochemical stability window, consequently boosting the specific capacity of battery [21]. Alternatively, raising the operating temperature will enhance the lithium-ion conductivity of the solid electrolyte and electrode reaction kinetics, which boosts battery rate capability. The improved thermodynamics introduces high mobility of Li ions and reduces activation polarization at the electrode, leading to a better realization of theoretical capacity. In addition, heat leads to larger nuclei radii, lower nucleation density, smoother lithium deposition, and softens the lithium to reduce risk associated with dendrite formation, as studied by Goodenough et al. [22]. However, the thermal stability of polymer is a big concern at high temperatures when it is used as a catholyte interlayer material. Therefore, the inclusion of a catholyte interlayer with an improved thermal and electrochemical stability is necessary, while carefully managing the amount of inactive material introduced to avoid reducing the capacity limit of ASSLBs [23].

In this study, we proposed the incorporation of triphenyl phosphate (TPP), a functional flame-retardant additive [24], into the PEO catholyte to introduce spontaneous fire-extinguishing properties, thus improving its stability for operating at high temperature. Furthermore, TPP enhances the thermal stability of the polymer catholyte at extreme temperatures, enabling it to recover its ability to safely and efficiently transfer Li^+^ ions after thermal abuse, in contrast to pristine polymer catholyte. Additionally, the inclusion of TPP maintains the thermal stability of interface, which suppresses undesirable side reactions that would otherwise lead to the consumption of active material NCM811 to impair the device performance. A garnet-based 4 V-Class ASSLBs using high-voltage cathode NCM811, LLZTO garnet electrolyte and a multi-functional PEO-TPP catholyte interlayer was fabricated which demonstrated enhanced cycling stability at elevated working temperature, superior safety performance in the aspect of flame retardancy, and comparable capacity performance to pristine PEO system.

## Experimental

### Preparation of Electrode and Electrolyte Material

The synthesis of NCM811 cathode and LLZTO solid-state electrolyte are detailed in Supporting Information. All assembly work was conducted within an argon-filled glove box. To fabricate full cells, the process began with the preparation of Li-Li_0.3_La_0.57_TiO_3_ (LLTO) composite anode by taking the procedure as described in our previous study to manage the variable introduced by the lithium anode metal and address potential issues arising from the anode-electrolyte interface [25]. This is done through continuous stirring of a molten mixture of Li and LLTO, maintaining a weight ratio of 95:5 in a stainless-steel crucible. The stirring was carried out for 15 min at 280 °C. Subsequently, an LLZTO (garnet) pellet was coated on one side with the Li-LLTO composite by immersing it in the molten anode composite.

To prepare the NCM cathode disks, the as-synthesized NCM811, polyvinylidene fluoride (PVDF), and Acetylene black (AB) were mixed at a weight ratio of 8:1:1 in N-methylpyrrolidone, and stirred for 40 min in vacuum to obtain the cathode slurry, which was then cast on aluminum foil, followed by vacuum drying at 120 °C overnight. The cathode foils were subsequently punched into 8 mm diameter discs, the areal loading of active material was approximately 2.0 mg·cm^−2^.

### Fabrication of PEO-TPP Catholyte Buffer Layer

The catholyte material is composed of PEO, TPP, and a Li^+^ conducting salt: lithium bis(trifluoromethane)sulfonimide (LiTFSI). Following our previous work on the construction of catholyte interlayer [18], 1 g of PEO, 0.434 g of LiTFSI (with a ratio of EO/Li^+^  = 15:1, n/n), and 0.2 g of TPP pellets (20 wt% of PEO) were combined through continuous stirring in 10 mL of acetonitrile at 60 °C overnight. Subsequently, a 50 μL portion of the liquid mixture was applied onto the surface of the LLZTO electrolyte. Afterward, Φ8 mm cathode disks were positioned on top, and the entire assembly was dried at 80 °C overnight inside the argon-filled glove box to ensure the thorough evaporation of the organic solvent.

**Fig. 1:**
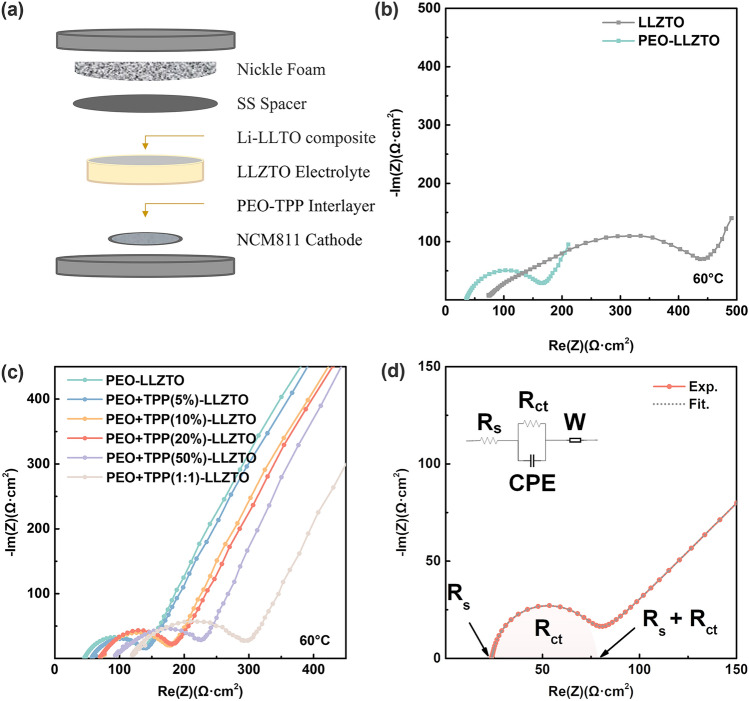
**a** General schematic of configuration of cell, Nyquist plots of **b** NCM|LLZTO|Li-LLTO full cells with and without PEO catholyte, **c** NCM|PEO-LLZTO|Li-LLTO full cells with and without different weight ratio of TPP functional additives, and **d** an example of experimental and fitted Nyquist plot using equivalent circuit model

The full cell was securely sealed within a CR2025 case, utilizing a Φ15 mm stainless steel plate and a Φ15 mm Ni foam as spacers and current collectors. The sealing process was executed using a specialized crippling machine (MTI, MSK-110). Details on the general schematic of the cell configuration are shown in Fig. 1a.

### Basic Characterization

The direct visualization of catholyte interlayer was observed by dual beam focussed ion beam scanning electron microscope (SEM, ZEISS NEON 40EsB FIBSEM). The distribution of elements across the electrode/interlayer/electrolyte was additionally examined through the utilization of an energy-dispersive X-ray spectroscope (EDS) integrated within the FIBSEM. The composition and organic structure of catholyte interlayer before and after galvanostatic cycling were examined by X-ray photoelectron spectroscopy (XPS) using a Kratos Axis Ultra-XPS with a hybrid lens and a 50 W monochromatic Al K_α_ (1486.6 eV) radiation source at 15 kV (10 mA), resolution levels of 160 and 40 nm are chosen for full (wide) spectra and high-resolution spectra, respectively. The raw data were analyzed using CasaXPS, with which the XPS spectra results were mathematically corrected and fitted using Shirley background and calibrated to the C–C/C–H peak (284.8 eV) from adventitious carbon contamination. X-ray diffraction (XRD) was employed to investigate the crystal structure and compositional information of catholyte interlayer before and after cycling, using a Bragg–Brentano geometry X-ray Diffractometer (Bruker D8A) with a copper X-ray source. Crystal structure of as synthesized and cycled cathode was observed and analyzed by FEI Talos FS200X G2 transmission electron microscope (TEM) with a field emission gun (FEG). The interplanar spacing is determined using ImageJ software and cross-referenced with the XRD results.

### Electrochemical Characterization

The lithium-ion transference number, *t*_Li+_, was determined electrochemically using a potentiostat BioLogic VSP and EC-lab in a Li-symmetrical cell, following the Bruce-Vincent method [26, 27]. To achieve this, potentiostatic polarization and EIS measurements were conducted. EIS was performed between 100 mHz and 200 kHz, both before and after potentiostatic polarization. The polarization was accomplished by applying a DC bias (Δ*V*) of 50 mV until the current reached a steady state (*I*_s_).

The transference numbers are determined via Eq. (1):

1 $${t}_{\text{Li}^{+}}=\frac{{I}_\text{s}\left(\Delta V-{{I}_{0}R}_\text{s}{\prime}\right)}{{I}_{0}\left(\Delta V-{{I}_\text{s}R}_{0}{\prime}\right)}$$

where *I*_0_ is the initial current, initial and steady state charge-transfer resistance are denoted as *R*_0_ and *R*_s_, respectively. Biologic VSP was also used to test the EIS of full batteries between 100 mHz and 100 kHz, as well as cyclic voltammetry (CV), linear sweep voltammetry (LSV), potentiostatic profile to validate the improved electrochemical stability of PEO-TPP catholyte interlayer. To assess the battery performance of the complete cells, a LANHE battery test system was employed to conduct galvanostatic Li plating and stripping (charge and discharge) under constant current density, which was calculated using the theoretical capacity of stoichiometric NCM811, i.e., 275.6 mAh·g^−1^. Additionally, the performance of the full cell was examined across different temperatures using a Thermoline Scientific laboratory oven fitted with a temperature controller.

## Results and Discussion

The importance of adding SPEs and increasing operation temperature on improving electrochemical performance of the ASSLBs was first confirmed by EIS. The Nyquist plot in Fig. S1 clearly demonstrates a substantial increase in the ionic conductivity of the SPEs at 60 °C as compared with that at RT. Figure 1b illustrates how the integration of the SPEs reduces interfacial resistance of LLZTO electrolyte, i.e., integration of PEO catholyte substantially reduces interfacial resistance substantially from 351.5 to 115.4 Ω·cm^2^ at 60 °C.

It was reported that some functional additives could facilitate the formation of cathode-electrolyte interphase (CEI) [28]. During electrochemical oxidation, such additive decomposes prior to the other electrolyte components to form a kinetically protective CEI layer, which suppresses further decomposition of the electrolyte at the electrode. Certain flame retardants (F- and P-based) used in LIBs can decompose at the electrode to form a protective layer in the same way to prevent thermal runaway [29–38]. For example, Cui et al. incorporated TPP into PVDF-HFP to substitute the conventional membrane separator in conventional LIBs, thereby introducing flame-retardant properties triggered by thermal decomposition of PVDF-HFP [39]. Recently, we have used liquid-state trimethyl phosphate (TMP) as wetting agent to realize highly stable anodic and cathodic interfaces with improved physical contact and chemical stability [35]. In this study, we selected TPP as an additive to enhance the electrochemical and thermal stability of the PEO-based catholyte considering its widespread use, cost effectiveness, and efficient performance as a phosphorus-based flame retardant [40]. With its distinct solid-state nature at RT, TPP plays a crucial role in tackling thermal stability challenge as a flame-retardant additive and contributes to the realization of an all-solid-state battery solution. As depicted in Fig. 1c, the introduction of TPP into SPEs results in an escalation of charge transfer resistance (*R*_ct_) at the interface, and the trend becomes more pronounced when TPP content reaches a mass ratio of 1:1 with PEO electrolyte. This phenomenon may be related to the high viscosity of TPP and its influence on the solvation of lithium-ion charge carriers [41], indicating the need for content optimization. It was found the obvious fire-retardant effect was demonstrated when TPP content is 20% or higher, which will be discussed in more detail later. The *R*_ct_ (charge transfer resistance at the interface) and *R*_s_ (bulk resistance of electrode and electrolyte material) of the electrolyte can be estimated by fitting the Nyquist plot with equivalent circuit model as demonstrated in Fig. 1d. Although the addition of 20 wt% TPP into PEO increased bulk resistance of the entire PEO-LLZTO system from 38.5 to 71.9 Ω·cm^2^, there is minimal effect on charge transfer resistance (107.3 Ω·cm^2^ for PEO-LLZTO and 119.5 Ω·cm^2^ for PEO/TPP-LLZTO) at 60 °C. Conversely, increasing TPP content to a 1:1 mass ratio with PEO electrolyte not only raises *R*_b_ from 38.5 to 119.6 Ω·cm^2^, but also results in a much larger *R*_ct_ (177.8 Ω·cm^2^) compared to pristine PEO-LLZTO. In light of achieving a suitable equilibrium between fire retardancy (as demonstrated in Fig. 5a) and interfacial resistance, we selected a composition of 20 wt% TPP within the PEO-TPP system as the optimized catholyte for subsequent experimental demonstrations.

**Fig. 2:**
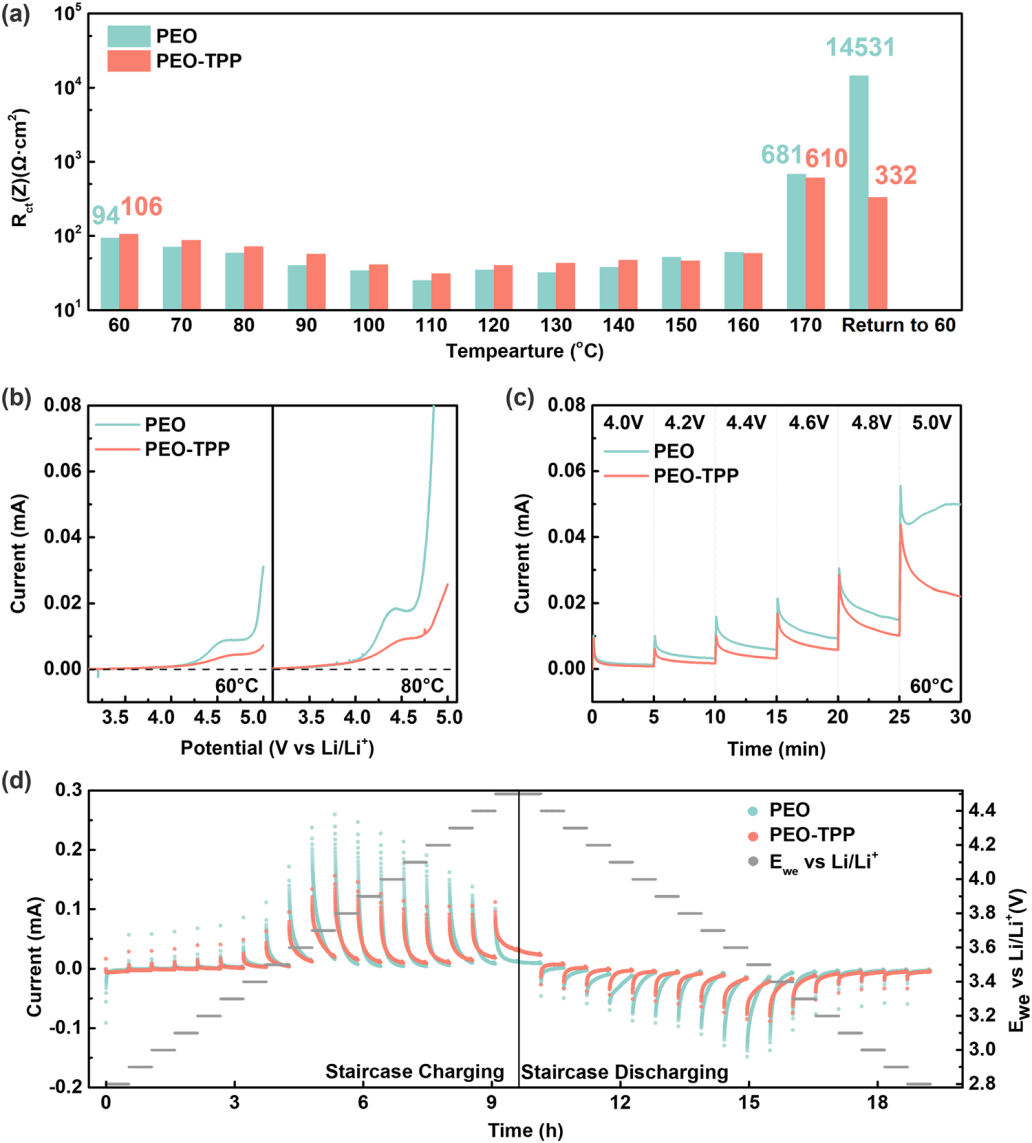
**a** Interfacial resistance derived from Nyquist plot of NCM|SPE-LLZTO|Li-LLTO when subject to thermal abuse up to 170 °C and corresponding thermal recovery, **b** LSV scanning from OCV to 5.0 V at 0.1 mV s^−1^ and **c** Potentiostatic Profile (step-wise) for PEO and PEO-TPP catholyte interlayer **d** Staircase Potentiostatic test from 2.8 to 4.5 V for PEO and PEO-TPP catholyte interlayer

The critical operation temperature for both PEO and PEO-TPP interlayers was found to be around 170 °C, at which point the catholyte experienced a significant decline in ionic conductivity based on the EIS results. While both systems exhibited a similar point of thermal failure, the PEO-TPP system had a satisfactory level of recovery after thermal stress. In contrast, the pristine PEO system displayed a substantial two orders of magnitude difference between its original and post-failure impedance, as shown in Fig. 2a and details of Nyquist plots in Fig. S2. This emphasizes TPP's inherent capability to offer greater thermal stability to the PEO-TPP system, rendering it more resilient against thermal degradation. It is widely acknowledged that phosphorus-based flame retardants generally operate by forming a protective film or barrier on the surface of combustible materials. This mechanism shields the material from the full extent of thermal runaway, a concept supported by earlier work [42]. In the case of TPP, previous studies have provided evidence for the formation of a char layer that acts as a protective film in the gaseous phase [43, 44]. Hence, the discovered thermal recovery of the PEO-TPP system may also be linked to a catalytic phenomenon, wherein TPP, functioning as a phosphate compound, initiated reactions that assisted in repairing thermal-induced impairments before the process of TPP gasification occurred. This could draw parallels to a condense phase mechanism of P-based flame retardants as well as their interactions and reactions with the surroundings polymeric material [45], providing a plausible explanation to the enhanced thermal recovery demonstrated by the PEO-TPP system compared to the unmodified PEO. Figure 2b, c displays measurements of LSV and Potentiostatic profiles, in which PEO-TPP shows a comparable oxidation potential but exhibits a reduced current response at the working electrode compared to pristine PEO. This observation indicates that the addition of TPP contributes to the suppression of PEO decomposition at high voltage. Staircase Potentiostatic (SP) test result aligns with this conclusion, where lower current response was obtained in most voltages applied to the PEO-TPP system (Fig. 2d).

**Fig. 3:**
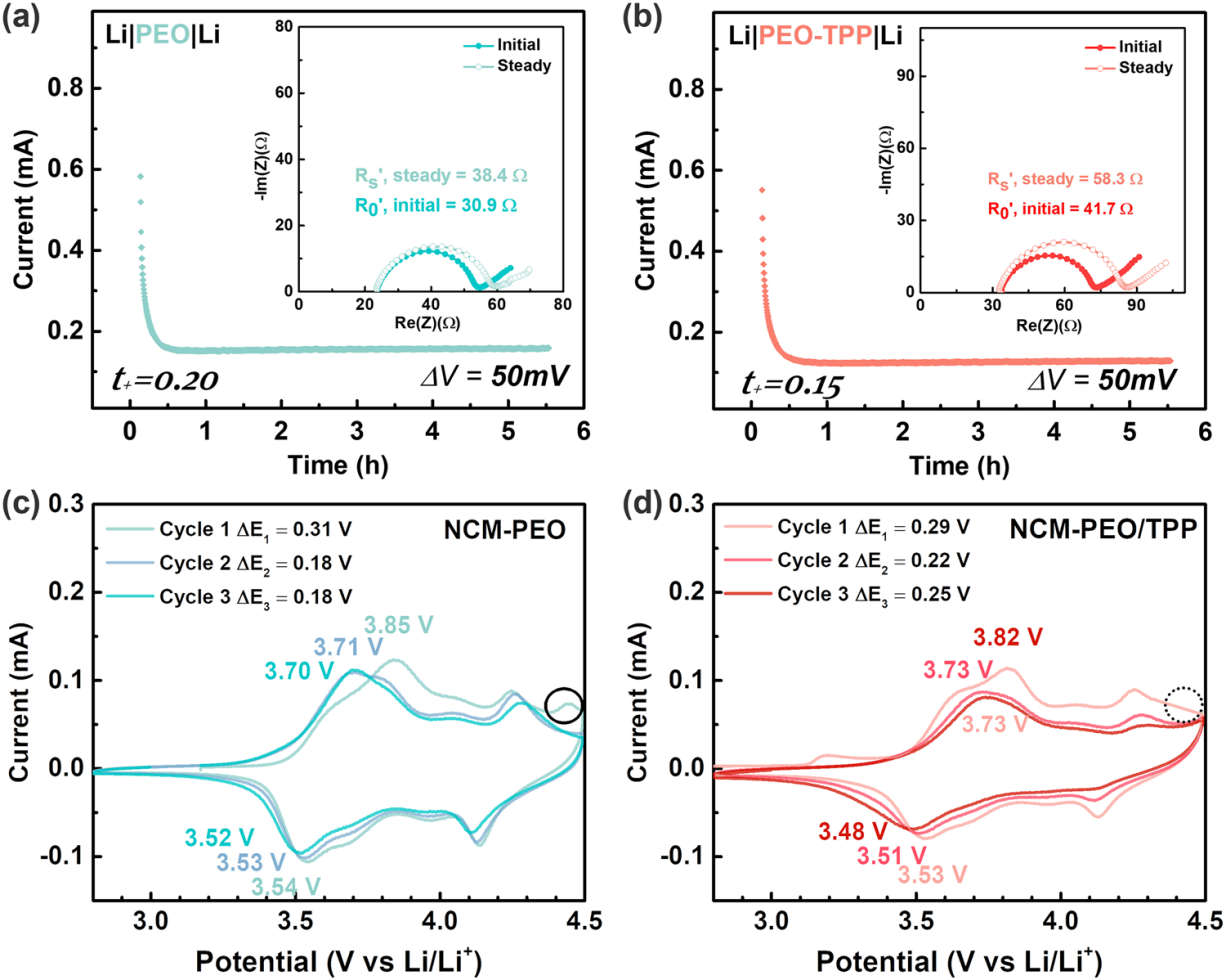
**a, b** Determination of Li^+^ transference number by symmetric polarization procedure at 60 °C. CV profile of **c** NCM811|PEO-LLZTO|Li-LLTO, **d** NCM811|PEO/TPP-LLZTO|Li-LLTO

The lithium-ion transference number, t_+_, represents the fraction of total ionic current carried by lithium ions during the battery's operation, which is crucial for assessing a battery's performance characteristics. Materials with high transfer numbers are desirable because they facilitate efficient lithium-ion transport, leading to better battery performance [46]. Figure 3a, b indicates that the PEO-TPP system demonstrated a relatively lower value of transference number (*t*_+_ = 0.15) as compared to pristine PEO (*t*_+_ = 0.20) Li^+^ transfer number at 60 °C, which undermines the Li^+^ mobility and aligns with the lower initial charge/discharge capacity, this consequently results in performance degradation due to electrode polarization. This adverse effect on lithium-ion transference number can be attributed to the difficulty of large PO_4_ anions hoping through the narrow ionic channel [47]. However, observation at the first anodic/cathodic peak in Fig. 3c, d indicates that PEO-TPP had little to negligible effect on reducing electrode polarization that stemmed from the irreversible structure change from phase transition of H1-M (i.e., Ni redox) [48]. Promisingly, the PEO-TPP system did exhibit some beneficial effect in alleviating oxygen evolution at 4.5 V, at which point the electrochemical breakdown of PEO-based SPE dominates the deterioration of interface [49]. The NCM-PEO/TPP system showed a smooth current response between 4.3 and 4.5 V, whereas the NCM-PEO system exhibited a noticeable peak that correlates to the oxidation of lattice oxygen through the following redox reactions [50], where *M* stands for transitional metal, i.e., Ni, Mn, and Co,

2 $$3\text{MO}_{2}\left(\text{layered}\right)\rightleftharpoons \text{M}_{3}\text{O}_{4}\left(\text{spinel}\right)+2\left[\text{O}\right]$$

3 $$\text{M}_{3}\text{O}_{4}\left(\text{spinel}\right)\rightleftharpoons 3\text{MO}\left(\text{rocksalt}\right)+\left[\text{O}\right]$$

This is attributed to the antioxidative property and high electrochemical stability of phosphate-based materials (both metal- and organo-phosphate) [51, 52], which contributed to the suppression of oxygen evolution reaction beyond 4.3 V as reported in previous researches [53, 54].

**Fig. 4:**
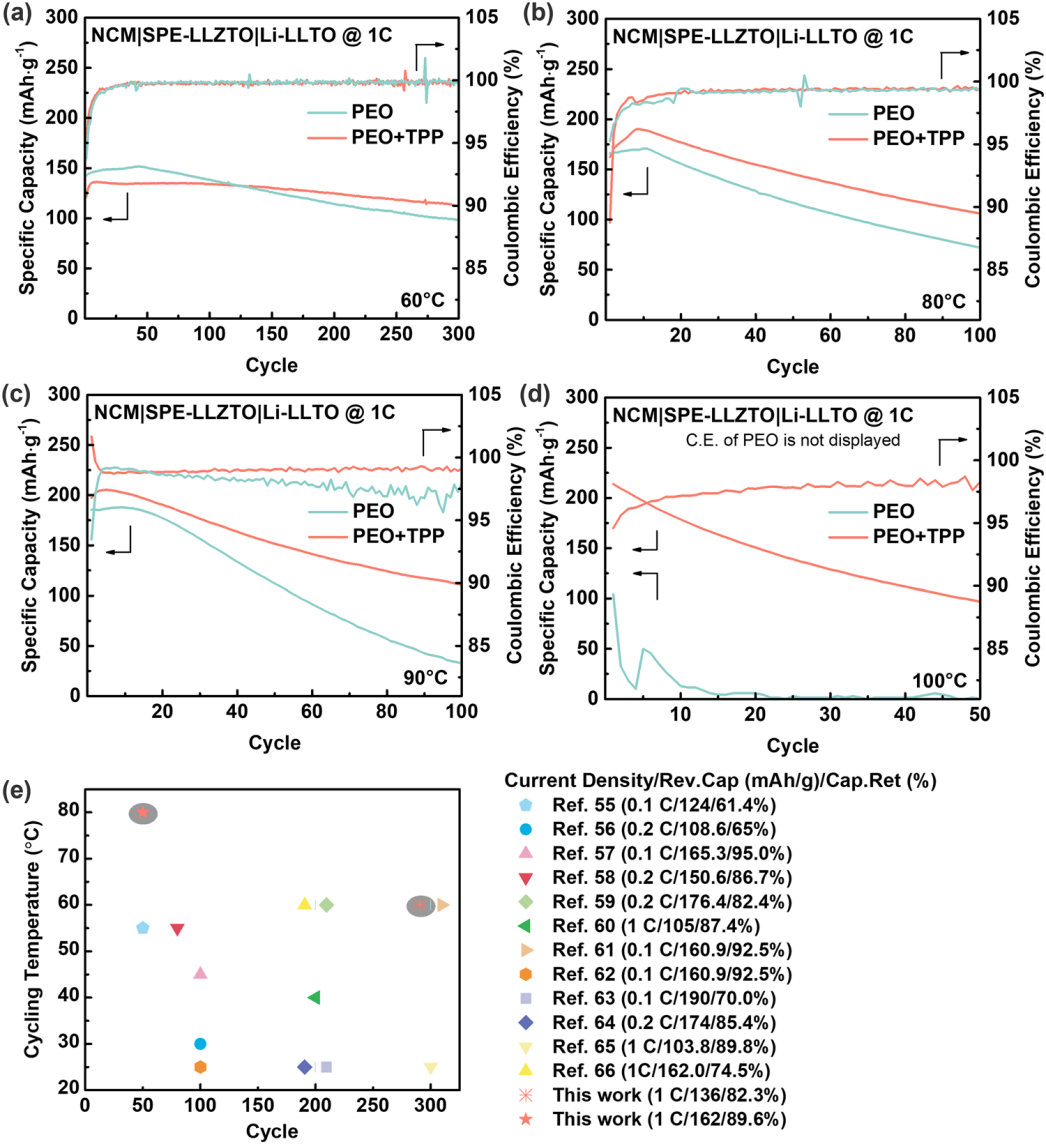
1 C charge/discharge full cell cycling performance for NCM|SPE-LLZTO|Li-LLTO at **a** 60 °C, **b** 80 °C, **c** 90 °C, and **d** 100 °C, where the choice of SPE are PEO and PEO-TPP, and **e** summary of initial discharge capacity as well as capacity retention after 50 cycles

Table 1 presents a comprehensive comparison of modified garnet-based ASSLBs featuring high-energy–density NCM cathodes, as reported over the last two years [55–66]. Although reduced Li^+^ transfer number by incorporation of TPP into the SPE was demonstrated previously, improved long-term charge/discharge stability was demonstrated in the aspect of cycling number, maximum operating temperature, and long-term stability. As shown in Fig. 4a, although cycling tests on PEO catholyte with the addition of TPP demonstrated lower initial discharge capacity, a consequence attributed to the expected rise in bulk resistance, the PEO-TPP system exhibited enhanced cycling stability at 60 °C, which became more pronounced at higher temperatures (Fig. 4b–d). The system also demonstrated safe cycling behavior up to 100 °C, despite the concurrent observation of heightened NCM cathode aging, with evidence from the charge/discharge profile in Fig. S3a, b [67]. Nonetheless, the presence of flame-retardant additives in PEO-TPP showed a higher degree of thermal stability, as a result the full cell cycling performance of the PEO-TPP system at various temperatures (60, 80, 90, and 100 °C) consistently outperformed that of the pristine PEO catholyte. It is particularly noteworthy that at 60 °C, a reversible capacity of 136.0 mAh·g^−1^ was achieved when charged at 1 C, accompanied by an impressive 98.5% capacity retention after 100 cycles. Additionally, it showcases an 89.6% capacity retention after 50 cycles when cycling at 80 °C. As a comparison, most of the reported NCM/LLZO-based ASSLBs are limited to a temperature range of 25–60 °C owing to the thermal instability of polymer catholyte or liquid-based wetting agent as shown in Fig. 4e. However, while achieving a capacity of 181.5 mAh·g^−1^ at a charging rate of 0.2 C, the capacity drops to less than 50 mAh·g^−1^ when charged at 3 C, as illustrated in Fig. S3c. This underscores the ASSLBs' mediocre rate capability, primarily attributed to the constraints imposed by low ionic conductivity.

**Table 1:** Comparison to recently reported performance of NCM/LLZO-based ASSLBs

Electrolyte	Cathode/anode	Operating potential (V)	Reversible capacity /current rate/cycle number/capacity retention	Temperature (°C)	References
Li_6.5_Mg_0.05_La_3_Zr_1.6_Ta_0.4_O_12_-PEO-TMOS-Pyr14FSI	NCM523/Li	3.0–4.3	124.0 mAh·g^−1^/0.1 C/50/61.4%	55	[55]
PEO-Li_6.4_La_3_Zr_1.4_Ta_0.6_O_12_-PAN	NCM111/Li	3.0–4.3	108.6 mAh·g^−1^/0.2 C/100/65%	30	[56]
Li_6.35_Ga_0.15_La_3_Zr_1.8_Nb_0.2_O_12_-SCN-3D	NCM523/Li (thin gold layer)	2.5–4.3	165.3 mAh·g^−1^/0.1 C/100/95.0%	45	[57]
Li_6.75_La_3_Zr_1.75_Ta_0.25_O_12_-PEO-PVDF-OX	Al_2_O_3_@NCM523/Li	2.5–4.3	150.6 mAh·g^−1^/0.2 C/80/86.7%	55	[58]
Li_7_La_3_Zr_2_O_12_-PEO	NCM622/Li	3.0–4.3	176.4 mAh·g^−1^/0.2 C/200/82.4%	60	[59]
Li_7_La_3_Zr_2_O_12_-LiBFSIE	NCM622 with liquid electrolyte/Li	2.5–4.2	113.0 mAh·g^−1^/0.6 C/200/NR	40	[60]
PEO-PVDF-LiF-Li_6.4_La_3_Zr_1.4_Ta_0.6_O_12_	NCM622 with LiBODFP layer/Li with PEO-3LiF-5LiDFOB layer	2.5–4.3	~ 105.0 mAh·g^−1^/1 C/300/87.4%	60	[61]
Li_6.25_Al_0.25_La_3_Zr_2_O_12_-PVDF-HFP-PAN	NCM811/Li	2.6–4.2	160.9 mAh·g^−1^/0.1 C/100/92.5%	RT	[62]
Li_6.4_La_3_Zr_1.4_Ta_0.6_O_12_-PEO-LiBOB-LiClO_4_	NCM811/Li	2.8–4.3	190.0 mAh·g^−1^/0.1 C/200/70.0%	25	[63]
Li_6.4_La_3_Zr_1.4_Ta_0.6_O_12_-PEGDA-SCN	NCM811/Li	2.5–4.3	174.0 mAh·g^−1^/0.2 C/200/85.4%	RT	[64]
Ga/F-Li_7_La_3_Zr_2_O_12_-PVDF-PAN	NCM811/ Li_2_MoO_4_/Li	2.7–4.2	103.8 mAh·g^−1^/1 C/300/89.8%	25	[65]
Li_6.4_La_3_Zr_1.4_Ta_0.6_O_12_-PEO-PVDF-LiTFSI-MgPFPAA	NCM811/Li	2.5–4.3	~ 162.0 mAh·g^−1^/1 C/200/74.5%	60	[66]
Li_6.4_La_3_Zr_1.4_Ta_0.6_O_12_ with PEO-TPP-LiTFSI Interlayer	NCM811/Li-LLTO	2.8–4.3	136.0 mAh·g^−1^/1 C/300/82.3%	60	This work
			162.0 mAh·g^−1^/1 C/50/89.6%	80

It's noteworthy to consider that, in theory, the initial discharge capacity should be proportional to temperature escalation due to change of thermal dynamics within the system, i.e., increasing temperature improves the specific capacity of the battery. Nevertheless, for the unmodified pristine PEO catholyte, this projected pattern did not persist at elevated temperatures (90 and 100 °C). In contrast, within the PEO-TPP catholyte system, this projected trend is consistently upheld, a phenomenon attributed to the augmented thermal stability encompassed by the inclusion of TPP.

**Fig. 5:**
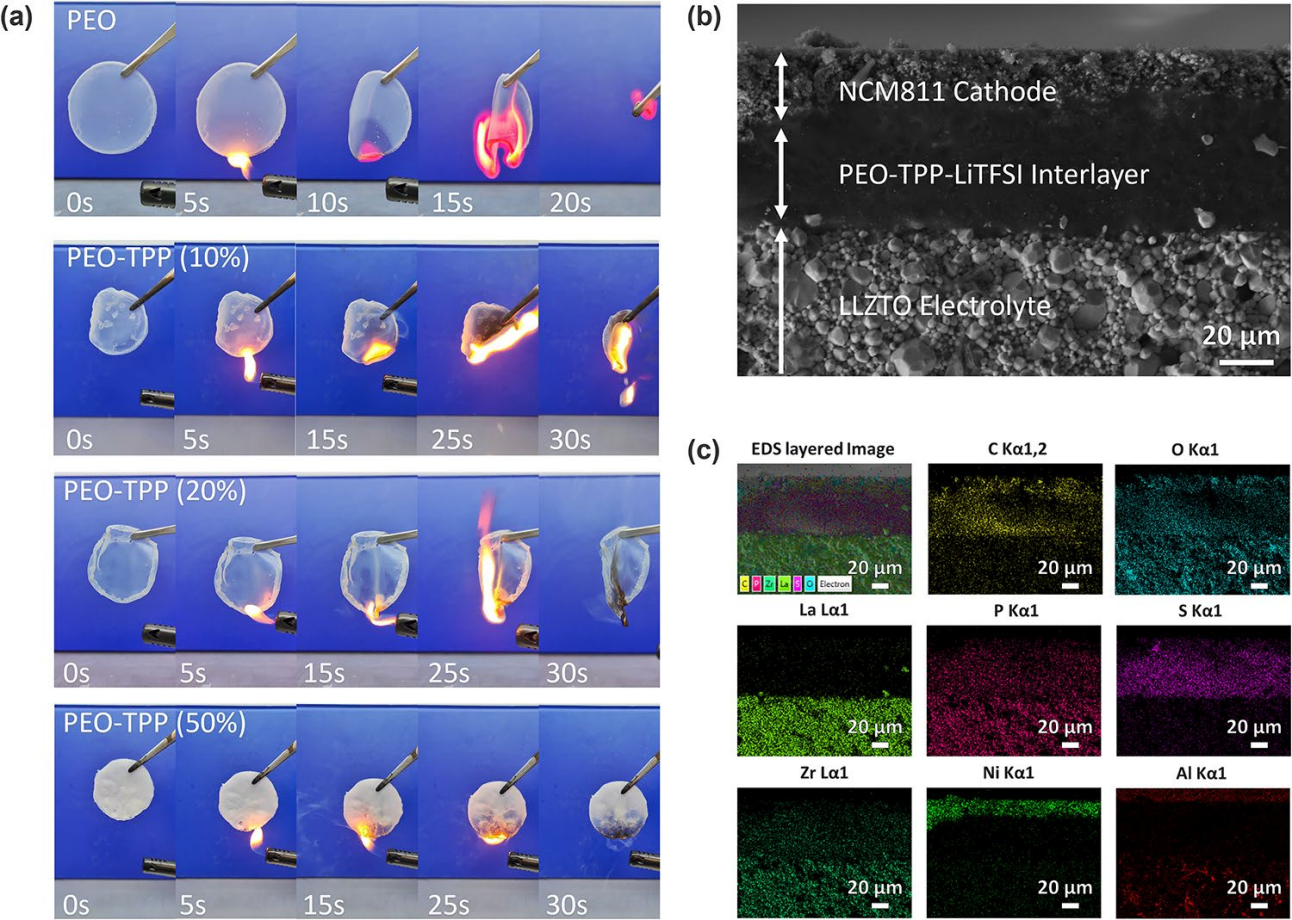
**a** Spontaneous fire-extinguishing ability of PEO-TPP via flammability test. **b** SEM cross-sectional image and **c** elemental mapping of the NCM811-PEO/TPP-LLZTO catholyte interlayer

The flammability test (Fig. 5a and Videos S1-S4) revealed the spontaneous fire-extinguishing ability of PEO-TPP. Increasing TPP concentration led to better prevention of combustion, indicating the effectiveness of TPP as a flame retardant. Specifically, both the pristine PEO and that with addition of 10% TPP were completely consumed by fire. But increasing the TPP concentration to 20% led to a notable difference as, when subjected to the flame, the spread of fire was stopped by the presence of TPP. For PEO and TPP with a weight ratio of 1:1, i.e., PEO-TPP (50%), combustion was effectively prevented by TPP in the first place, indicating its effectiveness as a flame retardant. However, higher TPP concentrations resulted in reduced flexibility of the polymer membrane, posing challenges in interlayer fabrication.

Observations from SEM–EDS analysis in Figs. 5b, c and S4 of the NCM811 cathode, PEO/TPP interlayer, and LLZTO electrolyte cross-section reveal the much-improved and distinctive surface contact, as well as even distribution of Li-conductive material across interfaces, facilitating Li ion diffusion. It is worth noting that the incorrect identification of phosphorus (P) in LLZTO electrolyte by EDS elemental mapping due to considerable overlap of P Kα 1 (2.014 keV) and P Kβ 1 (2.139 keV) with the strong signal of Zr Lα 1 (2.042 keV) (EDS, Fig. S5). This false signal was substantiated by the absence of carbon c and sulfur (S) in LLZTO, where carbon is originating from PEO and TPP, and sulfur is deriving from the lithium-conducting salt LiTFSI. These components collectively form the catholyte interlayer. Furthermore, trace amounts of aluminum (Al) were detected within the LLZTO electrolyte. This occurrence can be attributed to unavoidable contamination from the Al_2_O_3_ crucible utilized during the synthesis process. Regardless, the presence of Al is inconsequential to the functionality of the LLZTO electrolyte since Al was sometimes utilized as a dopant during the synthesis of LLZO to stabilize the cubic modification, and thus it should not yield any detrimental effects on the property of LLZTO electrolyte as confirmed in previous study [68].

**Fig. 6:**
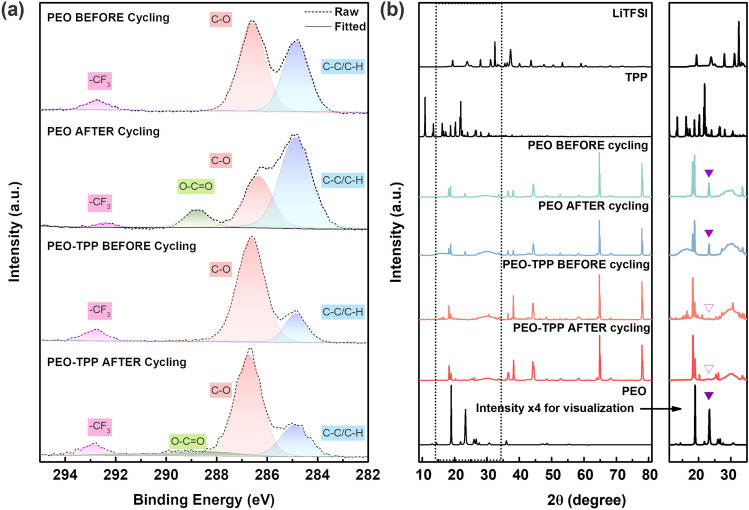
**a** C 1*s* XPS Spectra and **b** XRD pattern of NCM811/PEO interface, NCM811/PEO-TPP interface before and after cycling at 60 °C

To further understand the role of TPP additive in alleviating the effect of PEO degradation at elevated temperature, X-ray photoelectron spectroscopy (XPS) was conducted with the results shown in Fig. 6a. The XPS spectra were normalized to the CF_3_ related peak at 292.7 eV, as LiTFSI is typically more stable against electrochemical oxidation due to the strong TFSI anion [69, 70]. In NCM cathodes equipped with a catholyte interlayer composed of PEO, the appearance of the O–C=O peak within the C 1*s* spectra subsequent to cycling of 100 cycles is indicative of the oxidative degradation of PEO [71]. Additionally, a discernible reduction in C–O peak relative to C–C/C–H was observed, further validating the phenomenon of PEO degradation [49]. Conversely, when employing a PEO-TPP catholyte interlayer in NCM cathodes, only a vague signal corresponding to the O–C=O peak was identified in the C 1*s* spectra. It suggests the inclusion of the TPP additive effectively enhanced electrochemical stability by mitigating the degradation of the PEO catholyte under elevated working temperature and/or high electrochemical voltage. Nevertheless, an unidentified species was observed in the P 2*p* spectra on the shoulder of –PO_4_ peak, which may be attributed to the possible creation of CEI through the involvement of the –PO_4_ phosphate group stemming from the functional additive TPP (XPS and TEM, Figs. S6 and S7).

XRD pattern of the PEO and PEO-TPP systems before and after the cycling test were conducted to assess the crystalline nature of the polymer catholyte interlayers (Fig. 6b). The absence of characteristic peaks of TPP in cycled/uncycled catholyte interlayers indicates the complete dissolution of the TPP additive in PEO, owing to its exceptional solvating ability [72]. Concurrently, the inclusion of LiTFSI in the host polymer PEO significantly reduced peak intensity, in the meantime the interaction between the Li^+^ cation of the salt and the ether oxygen of PEO caused the peaks of PEO to shift to lower 2*θ* values [73]. Typically, incorporating the LiTFSI conducting agent into PEO leads to a reduction in the degree of crystallinity and an increase in ionic conductivity by suppressing the crystallization of PEO, an indicative observation is the decline of the characteristic peak at 19.4° and 23.7°. However, the complete absence of the 23.7° peak, in contrast to the pristine PEO interlayer, signifies the dominance of an amorphous phase that is possibly generated by the plasticization effect of TPP [74], potentially hindering the movement of polymer chains, leading to a reduction in the ionic conductivity of PEO interlayer which is confirmed by EIS result.

## Conclusion

In conclusion, we propose a solution to the challenges associated with the cathode-electrolyte interface by incorporating a multi-functional flame-retardant catholyte interlayer in garnet-based 4 V-Class ASSLBs to enable its operation at elevated temperature. The use of PEO catholyte enriched with TPP as the flame-retardant additive exhibited remarkable advancements in cycling stability and thermal safety at elevated temperatures, allowing for safe and stable cycling even at a high temperature of 100 °C, outperformed that of the pristine PEO catholyte. The PEO-TPP catholyte interlayer also displayed improved oxidative stability and better recovery from thermal abuse at 170 °C compared to the pristine PEO system. At 60 °C, the PEO-TPP system achieved a reversible capacity of 136.0 mAh·g^−1^ when charged at 1 C, with an impressive 98.5% capacity retention after 100 cycles.

Observations from SEM–EDS analysis of the NCM811 cathode, PEO/TPP interlayer and LLZTO electrolyte cross-section revealed improved surface contact and even distribution of Li-conductive material across interfaces, facilitating Li ion diffusion. XPS analysis further confirmed that TPP maintains the thermal stability of interface by suppressing the decomposition of PEO, curbing undesirable side reactions and preserving active material, thus enhancing the battery's overall performance. Flammability tests demonstrated the self-extinguishing property of the PEO-TPP system with TPP content exceeding 20%. The presented research not only advances the understanding of interface engineering in solid-state batteries but also offers practical insights for the design of high-performance, high-temperature batteries, paving the way for their broader implementation in various applications, from portable electronics to electric vehicles and large-scale energy storage. However, further enhancement in rate performance is imperative for the practical application of ASSLBs ensuring consistent capacity realization across a range of charge/discharge rates. Future work should prioritize the resolution of structural and kinetic limitations within the solid-state battery system to facilitate faster Li^+^ transport.

## Supplementary Information

Below is the link to the electronic supplementary material.

Supplementary file1 (MP4 7436 kb)

Supplementary file2 (MP4 11668 kb)

Supplementary file3 (MP4 8750 kb)

Supplementary file4 (MP4 18520 kb)

Supplementary file5 (PDF 1181 kb)
