# Supplementary material for: Enhanced High-Temperature Cycling Stability of Garnet-Based All Solid-State Lithium Battery Using a Multi-Functional Catholyte Buffer Layer
Source: Nanomicro Lett. 2024 Feb 19;16:124. doi: 10.1007/s40820-024-01358-9 (PMC10876510; doi:10.1007/s40820-024-01358-9)
Supplement: Supplementary file 5 — Supplementary file5 (PDF 1181 kb) [file 40820_2024_1358_MOESM5_ESM.pdf]

Supporting Information for

Enhanced High-Temperature Cycling Stability of Garnet-Based All Solid-State Lithium Battery Using a Multi-Functional Catholyte Buffer Layer

Leqi Zhao[1], Yijun Zhong[1], Chencheng Cao[1], Tony Tang[1], Zongping Shao[1,]*

[1]WA School of Mines: Minerals, Energy and Chemical Engineering, Curtin University, Perth, WA 6102, Australia

*Corresponding author. E-mail: zongping.shao@curtin.edu.au (Zongping Shao)

S1 Synthesis of cathode active material (CAM) [S1]

The transition-metal (TM) sulphates $NiSO_4{\cdot}6H_2O$ (≤100%, Sigma-Aldrich), $CoSO_4{\cdot}7H_2O$ (≤100%, Sigma-Aldrich), and $MnSO_4{\cdot}H_2O$ (≤100%, Sigma-Aldrich) in a molar ratio of 8:1:1 is weighed and dissolved in deionised water to form TM salt solution with a 2 M concentration. The as-prepared salt solution goes through a stirring reaction in which Ammonia solution (chelating agent, 4M NH_4OH) and sodium hydroxide solution (precipitating agent, 4 M NaOH) with stoichiometric amount of OH^- are slowly added till ideal pH range for co-precipitation is reached. The reaction environment is carefully controlled by temperature (55 °C), pH (11.5 – 11.7), atmosphere (inert, protected by N_2), stirring speed (500 rpm), and time (12-36 h) to prevent the formation of impurities such as MnOOH, MnO_2, Mn_3O_4.

After the reaction, the wet precipitates are washed with deionized water, filtered or centrifuged, and vacuum dried at 100 °C for 12 h to obtain the NCM precursor, i.e. $Ni_{0.8}Co_{0.1}Mn_{0.1}(OH)$. The NCM precursor is then dry mixed with Lithium hydroxide (10% excess). A two-stage calcination process is carried out in which the dried precipitates are exposed to pure oxygen flowing at a rate of 50 mL/min. The first stage of calcination is commonly known as the precursor formation step which helps to remove any residual organic compounds and initiates the formation of the desired oxide phases and is conducted at 480 °C for 5 h. This is followed by a second stage performed at 750 °C for 12 h, which is referred to as the crystallization step to ensure the formation and growth of well-crystallized and uniform particles and improves the homogeneity and electrochemical performance of the final cathode material.

S2 Synthesis of solid-state electrolyte (SSE) [S2]

LiOH (99.95%, Sigma-Aldrich), La_2O_3 (99%, Sigma-Aldrich), ZrO_2 (99%, Sigma-Aldrich), and Ta_2O_5 (99%, Sigma-Aldrich) in a molar ratio of 6.4:1.5:1.4:0.3 (with 10% excess of LiOH to compensate for calcination loss) is weighed and thoroughly mixed in a high energy ball mill with isopropanol as a solvent. La_2O_3 is heat-treated at 900 °C for 12 h in advance to remove potential carbonate impurities. After drying, the mixed powder undergoes a 900 °C calcination for 6 h in air to obtain the $Li_{6.4}La_3Zr_{1.4}Ta_{0.6}O_{12}$ (LLZTO) powder.

The as-prepared LLZTO powder is subjected to further milling/grinding prior to being pressed into pellets (D = 14 mm) under a pressure of 50 bar. A high-temperature sintering (1150 °C) is required to obtain the highly dense LLZTO garnet SSE, which is followed by surface polishing to achieve a thickness of 1 mm. The polished SSE pellets are to be stored in an argon-filled glove box to prevent surface contamination from CO_2 and H_2O.

S3 Supplementary Figures

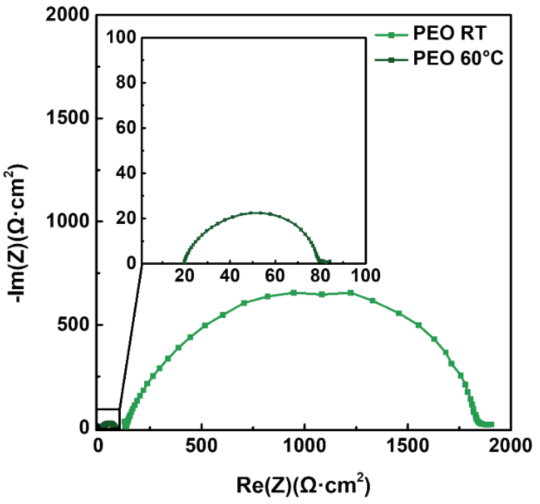

Fig. S1 Nyquist plots of Li|PEO|Li symmetric cell at room and elevated temperature

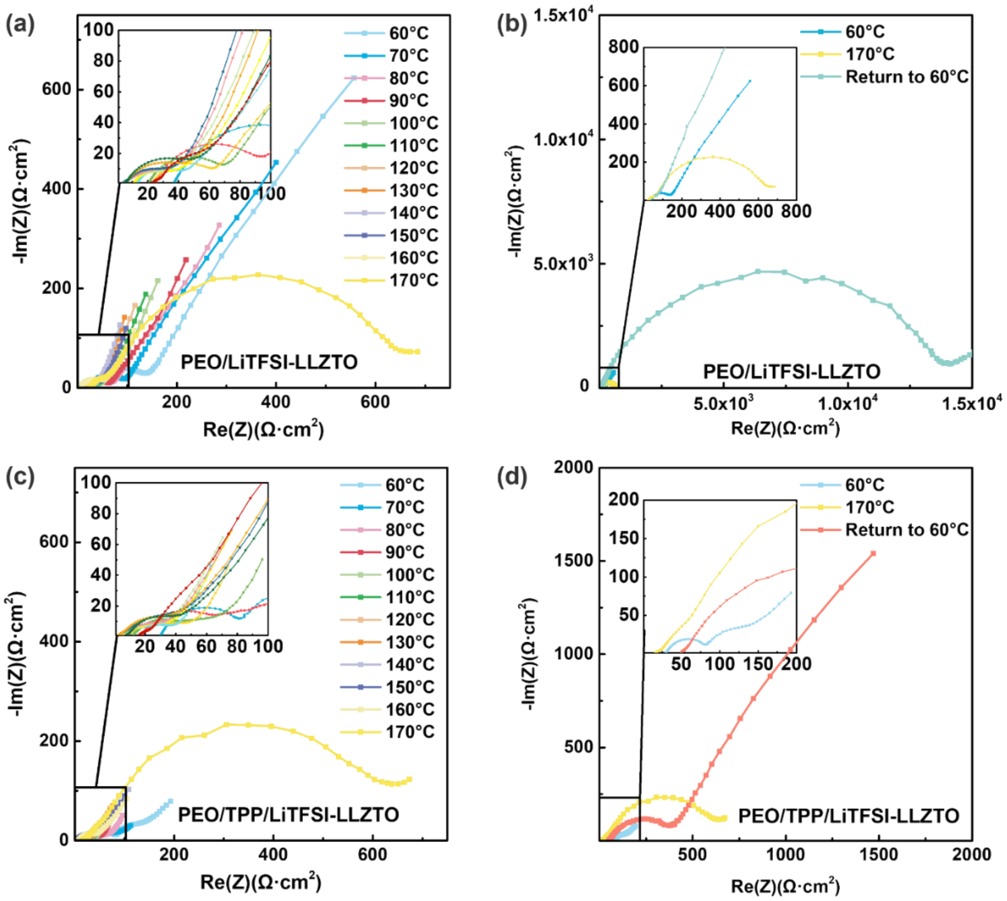

Fig. S2 Nyquist plots of full cell NCM|SPE-LLZTO|Li, revealing interface-level thermal stability determined via thermal abuse up to 170 °C for **a** PEO/LiTFSI-LLZTO and **c** PEO/TPP/LiTFSI-LLZTO. Recovery after thermal breakdown for **b** PEO/LiTFSI-LLZTO and **d** PEO/TPP/LiTFSI-LLZTO

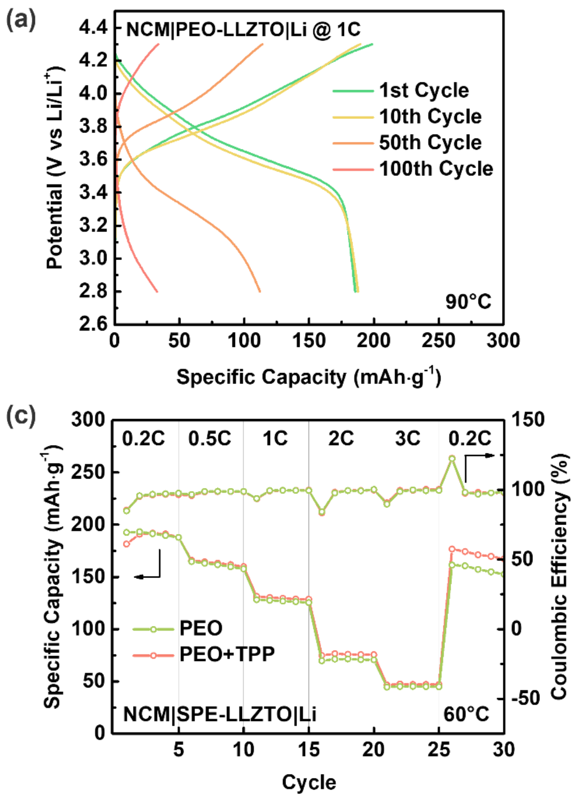

Fig. S3 Typical charge/discharge profile of **a** NCM|PEO-LLZTO|Li and **b** NCM|PEO/TPP-LLZTO|Li full battery at 1C, using 90 °C as an example, and **c** rate performance from 0.2C to 3C of NCM|SPE-LLZTO|Li at 60 °C

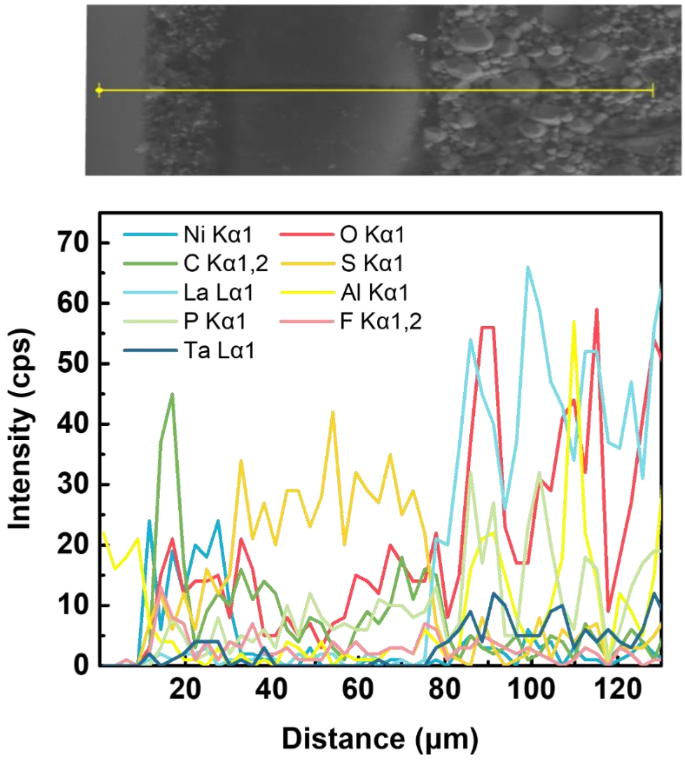

Fig. S4 Line scan (stacked) of element across cathode electrode interface of NCM-PEO/TPP-LLZTO

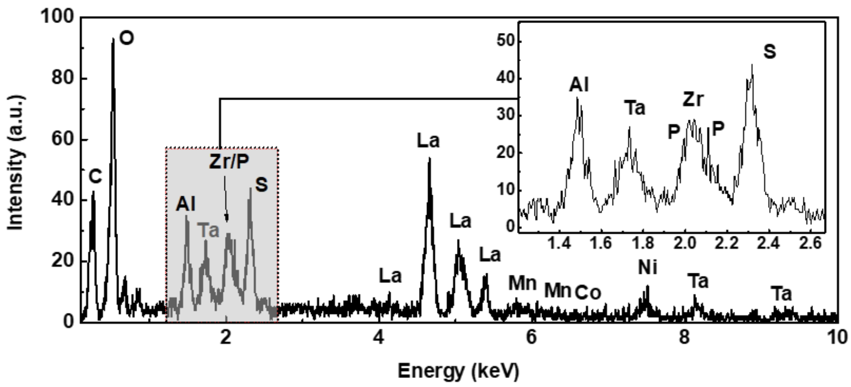

Fig. S5 EDS Spectrum detailing the overlap of P Kα 1 (2.014 keV) and P Kβ 1 (2.139 keV) with the strong signal of Zr Lα 1 (2.042 keV)

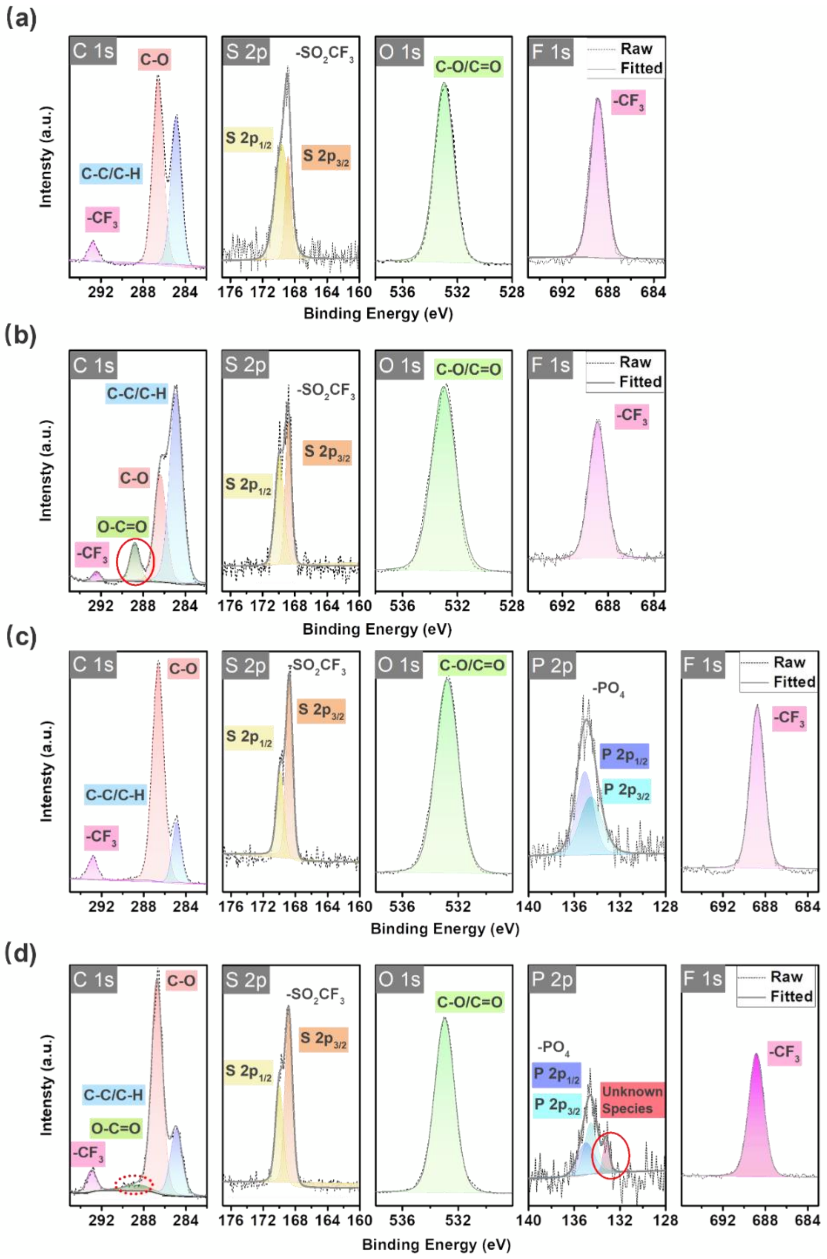

Fig. S6 XPS analysis of NCM811/PEO interface **a** before cycling and **c** after cycling, NCM811/PEO-TPP interface **b** before cycling and **d** after cycling at 60°C

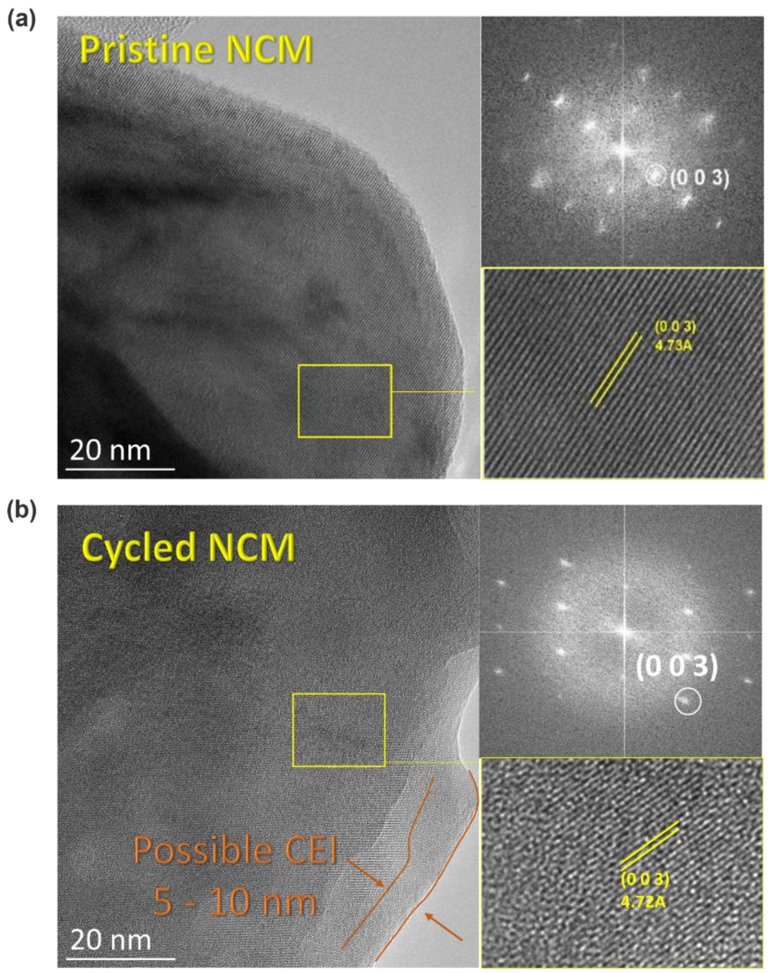

Fig S7. Transmission Electron Microscope (TEM) images of a) pristine and b) cycled NCM cathode for identifying the potential formation of CEI

Supplementary References

[S1] H. Zhang, J. Xu, and J. Zhang, Surface-coated $LiNi_{0.8}Co_{0.1}Mn_{0.1}O_2$ (NCM811) cathode materials by Al_2O_3, ZrO_2, and Li_2O-$2B_2O_3$ thin-layers for improving the performance of lithium ion batteries. Front. Mater. **6**, 00309 (2019). https://doi.org/10.3389/fmats.2019.00309

[S2] B. Liu, M. Du, B. Chen, Y. Zhong, J. Zhou, F. Ye, K. Liao, W. Zhou, C. Cao, R. Cai, and Z. Shao, A simple strategy that may effectively tackle the anode-electrolyte interface issues in solid-state lithium metal batteries. Chem. Eng. J. **427**, 131001 (2022). http://doi.org/10.1016/j.cej.2021.131001
